# The complex inflammatory indexes predict the prognostic risk for patients with acute coronary syndrome undergoing percutaneous coronary intervention

**DOI:** 10.1186/s12865-025-00745-0

**Published:** 2025-09-01

**Authors:** Ge Song, Yan Liu, Ying Zhang, Weichao Shan, Qiyu Sun, Yuewen Qi, Jingyi Liu, Lixian Sun

**Affiliations:** 1 Department of Cardiology, The Affiliated Hospital of Chengde Medical University, Chengde, 067000 China; 2 Hebei Key Laboratory of Panvascular Diseases, Chengde, 067000 China; 3 Department of Clinical Laboratory, The Affiliated Hospital of Chengde Medical University, Chengde, 067000 China; 4 Central Laboratory of Chengde Medical University Affiliated Hospital, Chengde, Hebei China

**Keywords:** Acute coronary syndrome, Neutrophil-to-lymphocyte * platelet ratio, Neutrophil-lymphocyte ratio, Percutaneous coronary intervention, Prognosis

## Abstract

**Introduction:**

An accurate assessment of prognostic risk is widely recognized to be important in improving the survival of patients with acute coronary syndrome (ACS). This study aimed to investigate the roles of neutrophil-to-lymphocyte * platelet (NLPR) and neutrophil-lymphocyte (NLR) ratios with high- and (HDL-C) and low-density lipoprotein cholesterol (LDL-C) levels in predicting the risk of major adverse cardiovascular events (MACEs) in patients with ACS undergoing percutaneous coronary intervention (PCI).

**Results:**

Overall, 1,263 patients with ACS undergoing PCI between January 2016 and December 2018 were consecutively enrolled. The patients were divided into MACEs (*n* = 54) and non-MACEs (*n* = 1,209) groups. The study endpoints were MACEs, including cardiac-related mortality and re-hospitalization for severe heart failure (HF), myocardial infarction (MI), and in-stent restenosis (ISR). The Kaplan–Meier curve showed the low NLPR and NLR groups had higher cumulative survival than the high NLPR and NLR group. Patients with high NLPR/HDL-C, NLPR×LDL-C, NLR/HDL-C, and NLR×LDL-C also had significantly lower cumulative survival.

**Conclusion:**

NLPR ≥ 2.843, NLPR/HDL-C ≥ 1.977, NLPR*LDL-C ≥ 4.608, NLR ≥ 0.025, NLR/HDL-C ≥ 0.030, and NLR*LDL-C ≥ 0.038 were all independent prognostic risk factors in patients with ACS undergoing PCI, which may be useful markers for long prognosis.

**Supplementary Information:**

The online version contains supplementary material available at 10.1186/s12865-025-00745-0.

## Introduction

Cardiovascular disease remains the leading cause of death worldwide, with nearly half of these deaths due to ischemic heart disease [1, 2]. Acute coronary syndromes (ACS) encompass a spectrum of conditions that include patients presenting with recent changes in clinical symptoms or signs, with or without changes on the 12-lead electrocardiogram (ECG), and with or without acute elevations in cardiac troponin (cTn) concentrations [3]. Myocardial revascularization via percutaneous coronary intervention (PCI) is the standard treatment for patients with ACS [4, 5]. 

An accurate assessment of prognostic risk is widely recognized to be important in improving the survival of patients with ACS [3]. In recent years, some researches have focused on the relationship between inflammation and atherosclerosis [6–9]. 

According to several clinical studies, the neutrophil-to-lymphocyte × platelet (NLPR) and neutrophil-lymphocyte (NLR) ratios as composite indicators of neutrophils, lymphocytes, and platelets, are novel markers reflecting the body’s immune response and inflammation level, and the prognosis of a wide range of disorders [10]. Increased NLPR and NLR are independent prognostic risk factors for overall survival in some diseases, for example, acute kidney injury, chronic calculus cholecystitis and Corona Virus Disease (COVID)‑19 [11–13]. 

Lipoprotein (a) is a risk factor for cardiovascular events [14]. Lipoprotein, especially high-density lipoprotein (HDL) and low-density lipoprotein (LDL) are closely related to cardiovascular disease (CVD) [15, 16]. 

The single inflammatory biomarkers, neutrophils, lymphocytes, platelets, HDL and LDL, are economical and convenient clinical lab indexes. However, the ability of novel complex indexes, NLPR and NLR combined with high-density lipoproteincholesterol (HDL-C) or low-density lipoproteincholesterol (LDL-C), as markers to predict prognostic risk in patients with ACS undergoing PCI, remains unclear. Therefore, the study aimed to investigate the values of NLPR, NLPR/HDL-C, NLPR×LDL-C, NLR, NLR/HDL-C, and NLR×LDL-C indexes in predicting the risk of major acute cardiovascular events (MACEs) in patients with ACS undergoing PCI.

## Methods

### Study design and population

A total of 1,263 patients with ACS who underwent PCI between January 2016 and December 2018 at the Affiliated Hospital of Chengde Medical University were consecutively enrolled in this study. All patients were treated according to international ACS practice guidelines [17]. 

The inclusion criteria were as follows: patients aged ⩾40 years; those with ACS (clinical types: unstable angina, non–ST elevation myocardial infarction, and ST-elevation myocardial infarction); those with coronary arteriography findings (the stenosis of at least 50% in one or more of the left main, left anterior descending, left circumflex, right coronary, or their main branches); and those who underwent PCI (complete revascularization) for the first time. The exclusion criteria were as follows: patients with coronary artery spasm and other secondary causes of angina or myocardial infarction, infectious diseases, malignant tumors, hematological diseases (such as anemia and leukopenia), severe heart diseases (such as aortic dissection and hypertrophic cardiomyopathy), severe systemic disease, chronic kidney disease (stage ⩾3), and incomplete blood cell count [18]. 

Patient data during hospitalization were collected by postgraduates who received professional training using standard procedures. Demographic and clinical characteristics and information regarding typical ACS clinical risk factors, such as diabetes, hypertension, dyslipidemia, and ischemic stroke, were collected during hospitalization. Hypertension was defined as systolic blood pressure ≥ 140 mm Hg (1 mm Hg = 0.133 kPa) and diastolic blood pressure ≥ 90 mm Hg at rest or a previous hypertension diagnosis with antihypertensive therapy. Diabetes mellitus was defined according to the following American Diabetes Association guidelines: a glycated hemoglobin value of ≥ 6.5%, a fasting plasma glucose value of ≥ 126 mg/dL (7.0 mmol/L), a 2 h plasma glucose value of ≥ 200 mg/dL (11.1 mmol/L) during an oral glucose tolerance test using 75 g of glucose, classic hyperglycemia symptoms (e.g., polyuria, polydipsia, and weight loss), or hyperglycemic crisis with a random plasma glucose value of ≥ 200 mg/dL (11.1 mmol/L). In the absence of unequivocal hyperglycemia, the first 3 criteria were confirmed by repeat testing. Dyslipidemia was defined as a serum total cholesterol value of ≥ 5.18 mmol/L (200 mg/dL), a high-density lipoprotein cholesterol value of ≤ 1.04 mmol/L, a low-density lipoprotein cholesterol value of ≥ 3.37 mmol/L, a triglyceride value of ≥ 1.7 mmol/L, or a previous dyslipidemia diagnosis with a prescribed medication. Experienced cardiologists performed PCI with the Judkins technique using 6 F right and left heart catheters. Procedural success was defined as a reduction in the percent diameter stenosis associated with thrombolysis to < 30% in myocardial infarction grades 2 or 3 [19]. All data were collected before discharge by the research team.

This study was approved by the Ethics Committee of the Affiliated Hospital of Chengde Medical University (approval number: CYFYLL2015006) and was conducted according to the tenets of the Declaration of Helsinki. All the participants provided informed consent.

#### Follow-up and endpoints

The follow-up data were collected via electronic medical records and/or clinic visits at 1, 3, 6, and 12 months, and annually thereafter. The primary study end points were MACEs, including cardiac-related mortality (death due to myocardial infarction, heart failure, fatal arrhythmia, and any other heart-related cause), re-hospitalization for severe heart failure (HF), myocardial infarction (MI), or in-stent restenosis (ISR).

##### Laboratory data

Fasting blood samples were collected within the first 24 h of admission before PCI. White blood cell (WBC), platelet, neutrophil, and lymphocyte counts were assessed using an automatic hematology analyzer (Sysmex XE-2100; Sysmex, Kobe, Japan). The HDL-C and LDL-C levels were measured using a Coulter Chemistry Analyzer (BECKMAN AU5800 Seri). The hematologic inflammatory markers were calculated as follows:

NLPR = neutrophils/lymphocytes × platelets.

NLPR/HDL-C = (neutrophils/lymphocytes × platelets)/high-density lipoprotein cholesterol.

NLPR×LDL-C = (neutrophils/lymphocytes × platelets) × low-density lipoprotein cholesterol.

NLR = neutrophils/lymphocytes.

NLR/HDL-C = (neutrophils/lymphocytes)/high-density lipoprotein cholesterol.

NLR×LDL-C = (neutrophils/lymphocytes) × low-density lipoprotein cholesterol.

###### Statistical analysis

The normality of the distribution of continuous variables was confirmed using the Kolmogorov–Smirnov test, and normally and non-normally distributed variables were presented as the mean ± standard deviation and as median with interquartile range, respectively. The differences in non-normally distributed continuous variables between the MACEs and non-MACEs groups were analyzed using the Mann–Whitney U test. Meanwhile, categorical variables were presented as numbers (%) and compared using the χ [2] test. Survival was estimated using the Kaplan–Meier method and compared between groups using the log-rank test. The diagnostic values of NLPR, NLPR/HDL-C, NLPR×LDL-C, NLR, NLR/HDL-C, and NLR×LDL-C were evaluated using receiver operating characteristic (ROC) curves, and the optimal cut-off value was determined using Youden’s index (sensitivity + specificity − 1). Significant variables in the univariate Cox proportional hazards model (i.e., those with *p* < 0.05) were entered into the multivariate Cox hazard proportional model. Additional interaction analyses were performed to determine possible effect modification by the subgroups. The R package time ROC and ggplot2 were used to plot the time-dependent ROC and area under the curve (AUC). The R package rms was used to plot the restricted cubic spline (RCS). All statistical analyses were performed using SPSS (version 26; SPSS Inc., Chicago, IL), GraphPad Prism 8.0 (GraphPad Software Inc., La Jolla, CA), and R 4.3.3; *p* < 0.05 was considered statistically significant.

## Results

### Patient characteristics

In total, 1,263 patients who completed the follow-up period were included in the final analysis. The median follow-up time was 1,014 days. Table 1 shows the characteristics of the patients in the MACEs (*n* = 54) and non-MACEs (*n* = 1,209) groups. The MACEs and non-MACEs groups showed significant differences in the proportion of patients with age ≥ 65 years (22 [36.7%] vs. 319 [24.2%]), history of HF (19 [31.7%] vs. 122 [9.3%]), unstable angina (UA) (14 [23.3%] vs. 516 [39.2%]), acute myocardial infarction (AMI) (46 [76.7%] vs. 801 [60.8%]), cardiogenic shock (6 [10.0%] vs. 15 [1.1%]), left ventricular ejection fraction (LVEF) < 40% (7 [11.7%] vs. 34 [2.6%]), NLPR ≥ 2.843 (29 [48.3%] vs. 292 [22.2%]), NLPR/HDL-C ≥ 1.977 (31 [51.7%] vs. 409 [31.1%]), NLPR×LDL-C ≥ 4.608 (34 [56.7%] vs. 458 [34.8%]), NLR ≥ 0.025 (29 [48.3%] vs. 292 [22.2%]), NLR/HDL-C ≥ 0.030 (31 [51.7%] vs. 409 [31.1%]), NLR×LDL-C ≥ 0.038 (34 [56.7%] vs. 458 [34.8%]) (all *p* < 0.05) (Table 1). The MACEs group was related with age ≥ 65 years, history of HF, AMI, cardiogenic shock, LVEF < 40%, NLPR ≥ 2.843, NLPR/HDL-C ≥ 1.977, NLPR*LDL-C ≥ 4.608, NLR ≥ 0.025, NLR/HDL-C ≥ 0.030, and NLR*LDL-C ≥ 0.038.

**Table 1:** Baseline patient characteristics of maces and non-MACEs groups

Variables	MACEs group (*n* = 54)	Non-MACEs group (*n* = 1209)	χ^2^/Z	*p*-value
**Demographic**				
Male	42 (70.0%)	982 (74.6%)	0.627	0.429
Age (years)	63.85 ± 8.46	58.73 ± 10.35	−3.581	< 0.001
Age ≥ 65 years	22 (36.7%)	319 (24.2%)	4.770	0.029
Dyslipidemia	34 (56.7%)	750 (56.9%)	0.002	0.996
Hypertension	33 (55.0%)	785 (59.6%)	0.505	0.477
Diabetes mellitus	16 (26.7%)	333 (25.3%)	0.058	0.810
History of HF	19 (31.7%)	122 (9.3%)	31.337	< 0.001
Family history of CAD	5 (8.3%)	197 (15.0%)	2.012	0.156
UA	14 (23.3%)	516 (39.2%)	6.087	0.014
AMI	46 (76.7%)	801 (60.8%)	6.087	0.014
Cardiogenic shock	6 (10.0%)	15 (1.1%)	30.003	< 0.001
**Laboratory data**				
WBC (10^9^/L)	9.39 ± 3.31	8.68 ± 3.27	−1.858	0.063
Platelet (10^9^/L)	203.18 ± 54.02	220.18 ± 56.72	−1.794	0.073
Neutrophil count (10^9^/L)	6.40 (4.39,9.40)	5.40 (3.95,8.00)	−2.296	0.022
Lymphocyte count (10^9^/L)	1.39 (0.94,1.96)	1.68 (1.24,2.29)	−2.719	0.007
Monocyte count (10^9^/L)	0.48 (0.33,0.75)	0.43 (0.32,0.57)	−1.771	0.076
HGB(g/L)	141.96 ± 16.36	146.78 ± 14.95	2.306	0.021
HGB ≤ 130 g/L	13 (24.1%)	177 (14.6%)	3.600	0.058
ALB (g/L)	38.82 ± 3.87	41.18 ± 3.56	4.763	< 0.001
ALB ≤ 35 g/L	7 (13%)	56 (4.8%)	0.599	0.439
Serum uric acid (µmol/L)	338.29 ± 87.97	329.92 ± 92.34	−0.653	0.514
Cr (µmol/L)	82.91 ± 31.50	69.11 ± 16.20	−5.794	< 0.001
Cr ≥ 110µmol/L	13 (24.1%)	304 (25.1%)	0.032	0.859
TC (mmol/L)	4.41 ± 1.12	4.43 ± 1.07	−0.025	0.980
TG (mmol/L)	1.42 (0.85,2.27)	1.60 (1.03,2.42)	−1.234	0.217
HDL (mmol/L)	1.15 ± 0.34	1.11 ± 0.30	−0.606	0.544
LDL (mmol/L)	2.41 ± 0.89	2.40 ± 0.84	−0.070	0.944
**Echocardiography**				
LVEDD > 50 mm	15 (25.0%)	330 (25.1%)	1.883	0.697
LVEF < 40%	7 (11.7%)	34 (2.6%)	16.396	<0.001
LVEF (%)	52.52 ± 10.12	57.17 ± 8.80	3.772	< 0.001
**Drugs**				
Aspirin (n, %)	48 (88.9%)	1202 (99.4%)	56.286	< 0.001
Clopidogrel (n, %)	38 (70.4%)	934 (77.3%)	1.381	0.240
Ticagrelor (n, %)	8 (14.8%)	268 (22.2%)	1.636	0.201
β-blocker (n, %)	29 (53.7%)	631 (52.2%)	0.047	0.828
ACEI/ARB (n, %)	21 (38.9%)	568 (47.0%)	1.360	0.244
Statins (n, %)	48 (88.9%)	1200 (99.3%)	47.337	< 0.001
Diuretic (n, %)	13 (24.1%)	83 (6.9%)	21.797	< 0.001
**Coronary angiography**				
1 vessel	14 (23.3%)	418 (31.7%)	1.883	0.170
2 vessels	19 (31.7%)	418 (31.7%)	0.000	0.991
3 vessels	27 (45.0%)	481 (36.6%)	1.771	0.183
**Indices**				
NLPR ≥ 2.843	29 (48.3%)	292 (22.2%)	21.970	< 0.001
NLPR/HDL-C ≥ 1.977	31 (51.7%)	409 (31.1%)	11.212	0.001
NLPR*LDL-C ≥ 4.608	34 (56.7%)	458 (34.8%)	12.837	< 0.001
NLR ≥ 0.025	29 (48.3%)	292 (22.2%)	21.970	< 0.001
NLR/HDL-C ≥ 0.030	31 (51.7%)	409 (31.1%)	11.212	0.001
NLR*LDL-C ≥ 0.038	34 (56.7%)	458 (34.8%)	12.837	< 0.001

#### Survival analysis and ROC curve

**Fig. 1:**
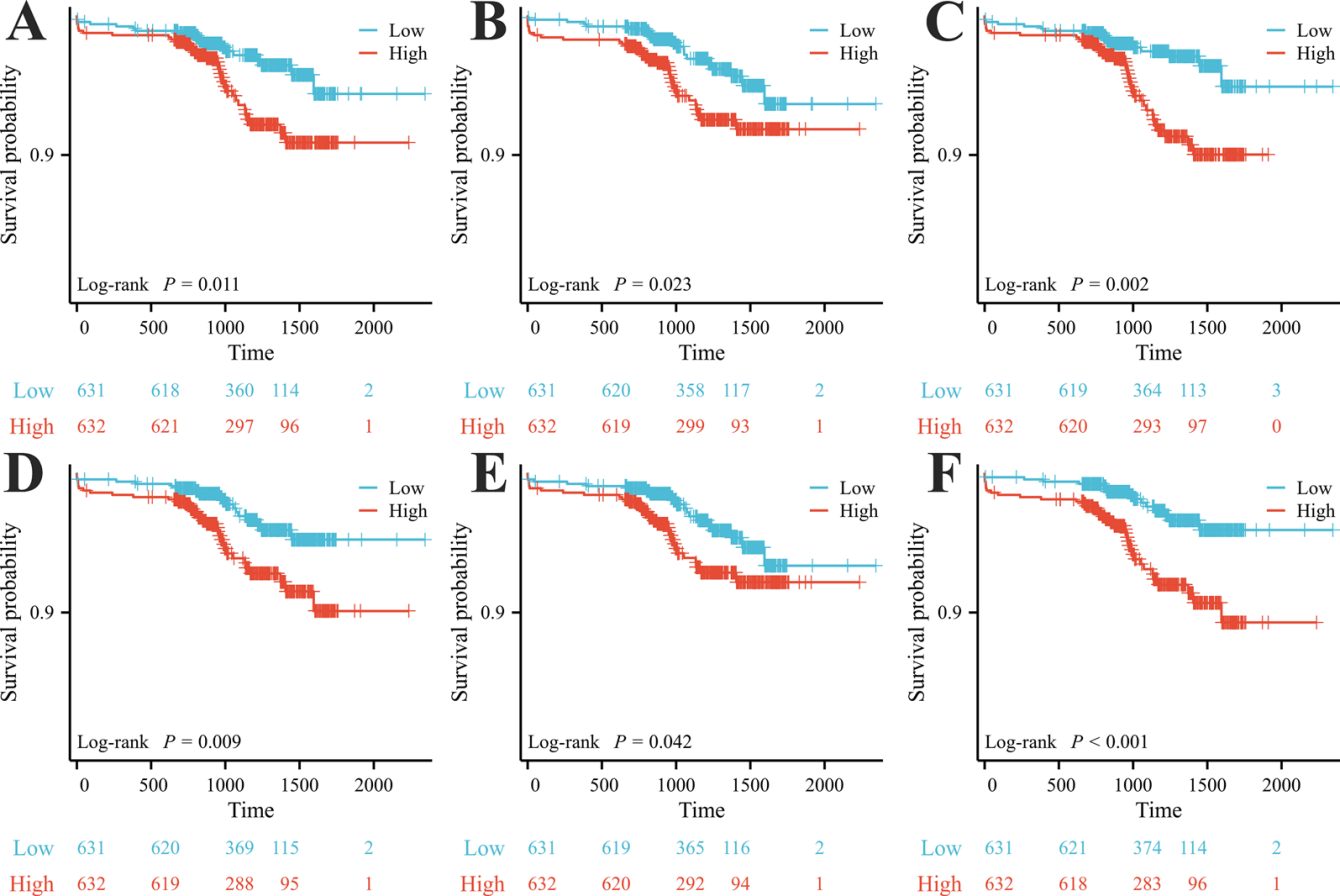
Kaplan–Meier (KM) curves of cumulative survival by NLPR, NLPR/HDL-C, NLPR×LDL-C, NLR, NLR/HDL-C, and NLR×LDL-C in ACS patients undergoing PCI (log-rank p). (A) KM curve of NLPR. (B) KM curve of NLPR/HDL-C. (C) KM curve of NLPR×LDL-C. (D) KM curve of NLR. (E) KM curve of NLR/HDL-C. (F) KM curve of NLR×LDL-C

The Kaplan–Meier curve (Fig. 1) showed that compared with the group with NLPR ≥ 2.843, the group of NLPR < 2.843 had higher cumulative survival, and the difference was statistically significant (log-rank *p* = 0.011). Similarly, high NLPR/HDL-C and NLPR×LDL-C levels also resulted in significantly lower cumulative survival (log-rank *p* = 0.023; log-rank *p* = 0.002). Compared with the group with NLR ≥ 0.025, the group of NLPR < 0.025 had higher cumulative survival, and the difference was statistically significant (log-rank *p* = 0.009). Similarly, high NLPR/HDL-C and NLPR×LDL-C levels also resulted in significantly lower cumulative survival (log-rank *p* = 0.042; log-rank *p* < 0.001). Table 2 presents the ROC curve analyses used to determine the optimal cut-off values for blood cell count-derived inflammation indices for the evaluation of MACEs in patients with ACS undergoing PCI. The AUC for NLPR was 0.646 (*p* < 0.001, 95% confidence interval [CI]: 0.568–0.723). The optimal diagnostic cut-off point was 2.843, with a sensitivity of 50.0% and specificity of 23.4%. The AUC for NLPR/HDL-C was 0.631 (*p* = 0.001; 95% CI: 0.551–0.711), and the optimal diagnostic cut-off point was 1.977, with a sensitivity of 53.7% and specificity of 31.9%. The AUC for NLPR×LDL-C was 0.635 (*p* = 0.001, 95% CI: 0.555–0.714), and the optimal diagnostic cut-off point was 4.608, with a sensitivity of 61.1% and a specificity of 36.4%. Similarly, the AUC for NLR was 0.631 (*p* = 0.001, 95% CI: 0.554–0.708). The optimal diagnostic cut-off point was 0.025, with a sensitivity of 53.7% and specificity of 30.2%. The AUC for NLR/HDL-C was 0.617 (*p* = 0.003; 95% CI: 0.539–0.696), and the optimal diagnostic cut-off point was 0.030, with a sensitivity of 46.3% and specificity of 22.2%. The AUC for NLR×LDL-C was 0.621 (*p* = 0.003, 95% CI: 0.545–0.698), and the optimal diagnostic cut-off point was 0.038, with a sensitivity of 70.4% and a specificity of 48.1%.

**Table 2:** ROC curve analyses of the blood cell count-derived inflammation indices between maces and non-MACEs groups

Indices	AUC (95%CI)	*p*-value	Se	Sp	Youden index	Cut-off
NLPR	0.646(0.568–0.723)	< 0.001	50.0%	23.4%	0.266	2.843
NLPR/HDL-C	0.631(0.551–0.711)	0.001	53.7%	31.9%	0.218	1.977
NLPR*LDL-C	0.635 (0.555–0.714)	0.001	61.1%	36.4%	0.247	4.608
NLR	0.631 (0.554–0.708)	0.001	53.7%	30.2%	0.235	0.025
NLR/HDL-C	0.617 (0.539–0.696)	0.003	46.3%	22.2%	0.241	0.030
NLR*LDL-C	0.621 (0.545–0.698)	0.003	70.4%	48.1%	0.222	0.038

##### Time-dependent ROC and AUC

**Fig. 2:**
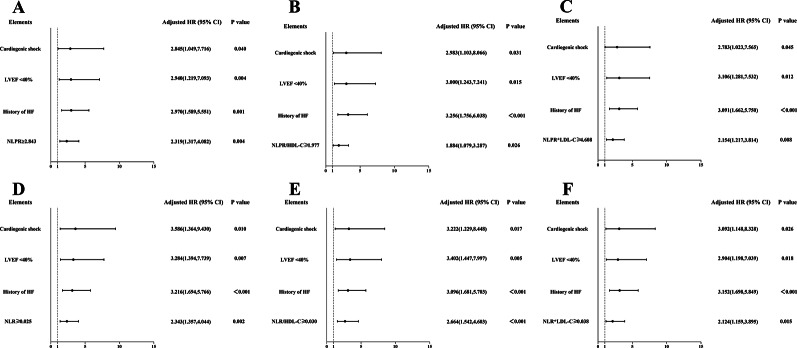
Time-dependent receiver operating characteristic (ROC) curves between MACEs of different inflammatory indexes. (A) time-dependent ROC curve of NLPR. (B) time-dependent ROC curve of NLPR/HDL-C. (C) time-dependent ROC curve of NLPR×LDL-C. (D) time-dependent ROC curve of NLR. (E) time-dependent ROC curve of NLR/HDL-C. (F) time-dependent ROC curve of NLR×LDL-C. (G) Time-dependent AUC curves between different MACEs of different inflammatory indexes

**Fig. 4:**
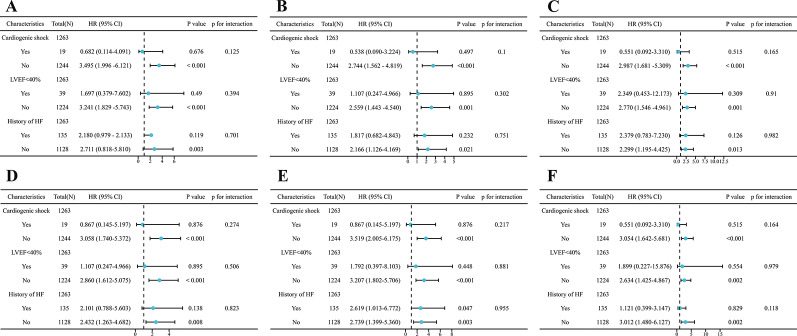
Subgroup analysis and P for interaction. (A) Model 1 is for NLPR. (B) Model 2 is for NLPR/HDL-C. (C) Model 3 is for NLPR×LDL-C. (D) Model 4 is for NLR. (E) Model 5 is for NLR/HDL-C. (F) Model 6 is for NLR×LDL-C

Figure 2 shows the time-dependent ROC of NLPR (Fig. 2A), NLPR/HDL-C (Fig. 2B), NLPR×LDL-C (Fig. 2C), NLR (Fig. 2D), NLR/HDL-C (Fig. 2E), and NLR×LDL-C (Fig. 2F). In NLPR group, the 1-, 2-, 3-, 4-, and 5-year AUCs were 0.648, 0.629, 0.673, 0.670, and 0.751, respectively (Fig. 2G). In NLR group, the 1-, 2-, 3-, 4-, and 5-year AUCs were 0.657, 0.634, 0.660, 0.645, and 0.623, respectively (Fig. 2G). As shown in Fig. 4, the AUC of NLPR, NLPR/HDL, and NLPR×LDL-C and 95% CI tended to increase with time. The time-dependent AUC showed an almost increasing tendency, suggesting that the diagnostic efficiency of NLPR, NLPR/HDL, and NLPR×LDL-C increased with time.

###### Univariate and multivariate Cox hazard proportional models

The univariate Cox proportional hazard model showed that a high NLPR (≥ 2.843) was an independent risk factor for patients with ACS undergoing PCI (hazard ratio [HR], 3.269; 95% CI: 1.917–5.537, *p* < 0.001). A high NLPR/HDL-C (≥ 1.977) and a high NLPR×LDL-C (≥ 4.608) were also independent risk factors for patients with ACS undergoing PCI (HR: 2.532, 95% CI: 1.483–4.325, *p* = 0.001; and HR: 2.883, 95% CI: 1.668–4.985, *p* < 0.001, respectively). A high NLR (≥ 0.025) was an independent risk factor for patients with ACS undergoing PCI (HR: 2.838; 95% CI: 1.661–4.849, *p* < 0.001). A high NLR/HDL-C (≥ 0.030) and a high NLR×LDL-C (≥ 0.038) were also independent risk factors for patients with ACS undergoing PCI (HR: 3.317, 95% CI: 1.940–5.670, *p* < 0.001; and HR: 2.825, 95% CI: 1.574–5.071, *p* = 0.001, respectively).

**Fig. 3:**
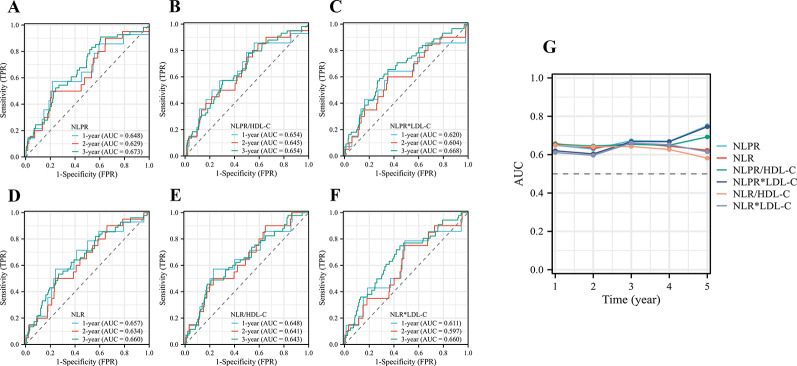
Forest graphs according to Cox proportional hazards regression model to test the risk factors for MACEs. (A) Model 1 is adjusted for NLPR ≥ 2.843, history of HF, LVEF < 40%, and cardiogenic shock. (B) Model 2 is adjusted for NLPR/HDL-C ≥ 1.977, history of HF, LVEF < 40%, and cardiogenic shock. (C) Model 3 is adjusted for NLPR×LDL-C ≥ 4.608, history of HF, LVEF < 40%, and cardiogenic shock. (D) Model 4 is adjusted for NLR ≥ 0.025, history of HF, LVEF < 40%, and cardiogenic shock. (E) Model 5 is adjusted for NLR/HDL-C ≥ 0.030, history of HF, LVEF < 40%, and cardiogenic shock. (F) Model 6 is adjusted for NLR×LDL-C ≥ 0.038, history of HF, LVEF < 40%, and cardiogenic shock

Other significant factors were cardiogenic shock (*p* < 0.001), LVEF < 40% (*p* < 0.001), and history of HF (*p* < 0.001). Therefore, cardiogenic shock, LVEF < 40%, history of HF, and indices were finally selected in the multivariate Cox proportional hazards model through adjusted variables, and variables that effectively influenced the prognosis of patients with ACS were selected. After adjustment for cardiogenic shock, LVEF < 40%, and history of HF, the multivariate Cox proportional hazard model showed that NLPR ≥ 2.843, NLPR/HDL-C ≥ 1.977, NLPR×LDL-C ≥ 4.608, NLR ≥ 0.025, NLR/HDL-C ≥ 0.030, and NLR×LDL-C ≥ 0.038 were independent risk factors for patients with ACS undergoing PCI: NLPR ≥ 2.843 (HR: 2.319, 95% CI: 1.317–4.082, *p* = 0.004), cardiogenic shock (HR: 2.845, 95% CI: 1.049–7.716, *p* = 0.040), LVEF < 40% (HR: 2.940, 95% CI: 1.219–7.093, *p* = 0.004), history of HF (HR: 2.970, 95% CI: 1.589–5.551, *p* = 0.001) (Table 3; Fig. 3A); NLPR/HDL-C ≥ 1.977 (HR: 1.884, 95% CI: 1.079–3.287, *p* = 0.026), cardiogenic shock (HR: 2.983, 95% CI: 1.103–8.066, *p* = 0.031), LVEF < 40% (HR: 3.000, 95% CI: 1.243–7.241, *p* = 0.015), history of HF (HR: 3.256, 95% CI: 1.756–6.038, *p* < 0.001) (Table 3; Fig. 3B); NLPR×LDL-C ≥ 4.608 (HR: 2.154, 95% CI: 1.217–3.814, *p* = 0.008), cardiogenic shock (HR: 2.783, 95% CI: 1.023–7.565, *p* = 0.045), LVEF < 40% (HR: 3.106, 95% CI: 1.281–7.532, *p* = 0.012), history of HF (HR: 3.091, 95% CI: 1.662–5.750, *p* < 0.001) (Table 3; Fig. 3C); NLR ≥ 0.025 (HR: 2.343, 95% CI: 1.357–4.044, *p* = 0.002), cardiogenic shock (HR: 3.586, 95% CI: 1.364–9.430, *p* = 0.010), LVEF < 40% (HR: 3.284, 95% CI: 1.394–7.739, *p* = 0.007), history of HF (HR: 3.216, 95% CI: 1.694–5.766, *p* < 0.001) (Table 3; Fig. 3D); NLR/HDL-C ≥ 0.030 (HR: 2.664, 95% CI: 1.542–4.603, *p* < 0.001), cardiogenic shock (HR: 3.222, 95% CI: 1.229–8.448, *p* = 0.017), LVEF < 40% (HR: 3.402, 95% CI: 1.447–7.997, *p* = 0.005), history of HF (HR: 3.096, 95% CI: 1.681–5.703, *p* < 0.001) (Table 3; Fig. 3E); NLR×LDL-C ≥ 0.038 (HR: 2.124, 95% CI: 1.159–3.895, *p* = 0.015), cardiogenic shock (HR: 3.092, 95% CI: 1.148–8.328, *p* = 0.026), LVEF < 40% (HR: 2.904, 95% CI: 1.198–7.039, *p* = 0.018), history of HF (HR: 3.152, 95% CI: 1.698–5.849, *p* < 0.001) (Table 3; Fig. 3F).

**Table 3:** Cox hazard proportional models of maces risk according to blood cell count-derived inflammation indices

Indices	Model 1		Model 2	
	HR (95%CI)	*p*-value	HR (95%CI)	*p*-value
NLPR	3.269 (1.917–5.537)	<0.001	2.319 (1.317–6.038)	0.004
NLPR/HDL-C	2.532 (1.483–4.325)	0.001	1.884 (1.079–3.287)	0.026
NLPR*LDL-C	2.883 (1.668–4.985)	<0.001	2.154 (1.217–3.814)	0.008
NLR	2.838 (1.661–4.849)	<0.001	2.343 (1.357–4.044)	0.002
NLR/HDL-C	3.317 (1.940–5.670)	<0.001	2.664 (1.542–4.603)	<0.001
NLR*LDL-C	2.825 (1.574–5.071)	0.001	2.124 (1.159–3.895)	0.015

Model 1: Unadjusted

Model 2: Adjusted for history of HF, LVEF < 40%, and cardiogenic shock

####### Subgroup analysis

In order to study the risk and interaction of inflammatory markers in people with ACS undergoing PCI, we further analyzed three subgroups of NLPR, NLPR/HDL-C, NLPR×LDL-C, NLR, NLR/HDL-C, and NLR×LDL-C and ACS risk indicators (cardiogenic shock, LVEF < 40%, and history of HF). Figure 4 shows that cardiogenic shock, LVEF < 40%, and history of HF did not modify the association between NLPR, NLPR/HDL-C, NLPR×LDL-C, NLR, NLR/HDL-C, and NLR×LDL-C and the risk of ACS in patients undergoing PCI.

######## Restricted cubic spline

**Fig. 5:**
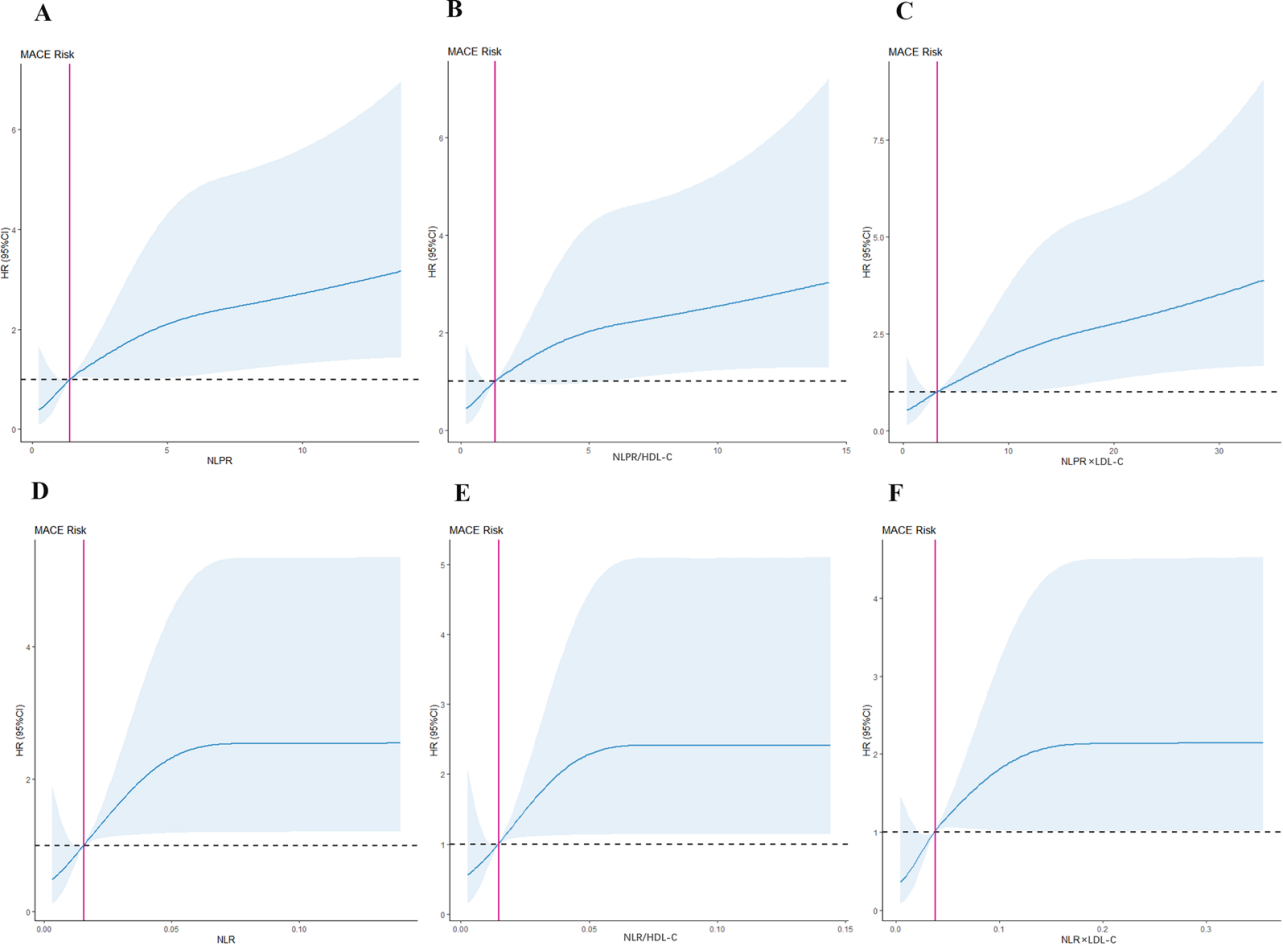
Restricted cubic spline (RCS) models. (A) Model 1 is for NLPR. (B) Model 2 is for NLPR/HDL-C. (C) Model 3 is for NLPR×LDL-C. (D) Model 4 is for NLR. (E) Model 5 is for NLR/HDL-C. (F) Model 6 is for NLR×LDL-C

The RCS models were generated to visualize the relationships between NLPR, NLPR/HDL-C, NLPR×LDL-C, NLR, NLR/HDL-C, and NLR×LDL-C, and prognostic risk. Model 1 (Fig. 5A) was used for NLPR, Model 2 (Fig. 5B) for NLPR/HDL-C, Model 3 (Fig. 5C) for NLPR×LDL-C, Model 4 (Fig. 5D) for NLR, Model 5 (Fig. 5E) for NLR/HDL-C, and Model 6 (Fig. 5F) for NLR×LDL-C. The risk of developing ACS in patients undergoing PCI increased with advanced systemic inflammation-response index (SIRI), SIRI/HDL-C, and SIRI×LDL-C levels. As shown, high NLPR, NLPR/HDL-C, NLPR×LDL-C, NLR, NLR/HDL-C, and NLR×LDL-C were independent risk factors.

## Discussion

The main findings of this study were as follows. First, NLPR ≥ 2.843, NLPR/HDL-C ≥ 1.977, NLPR×LDL-C ≥ 4.608, NLR ≥ 0.025, NLR/HDL-C ≥ 0.030, and NLR×LDL-C ≥ 0.038, as independent risk factors, were correlated with poor prognosis for patients with ACS undergoing PCI. Second, the value of the novel indexes, NLPR, NLPR/HDL-C, NLPR×LDL-C, NLR, NLR/HDL-C, and NLR×LDL-C had almost the same ability to predict prognosis in patients with ACS who underwent PCI as classical indicators like cardiogenic shock, history of HF and LVEF < 40%. Finally, the HR trend of patients with ACS undergoing PCI was non-linear; however, increased gradually with NLPR, NLPR/HDL-C, NLPR×LDL-C, NLR, NLR/HDL-C, and NLR×LDL-C values. To the best of our knowledge, this is the first study to analyze the correlation between this novel index, NLPR and NLR with HDL-C or LDL-C, and the prognosis of patients with ACS who underwent PCI.

The NLR, as an indicator of ozone exposure, was first developed in 1967 [20]. NLR is an easily measured and cheap parameter of systemic inflammation. Lipoprotein (a) is a risk factor for cardiovascular events, especially HDL-C and LDL-C [14].– [15] LDL-C levels are associated with the risk of cardiovascular disease [16]. By combining the inflammatory biomarkers with HDL-C and LDL-C, a previous study derived new indexes and proved that they had higher diagnostic efficiency for coronary heart disease [21]. However, the values of the composite novel indexes of NLPR, NLPR/HDL-C, NLPR×LDL-C, NLR, NLR/HDL-C, and NLR×LDL-C on the prognosis of ACS remains unknown.

NLR has been found to be a significant predictor of cardiovascular risk, kidney illness, cancers, and so on. The elevated NLR on admission in patients with acute HF was independently associated with worse cardiovascular events, re-hospitalization for HF, in-hospital death, and composite outcomes [22]. NLR was also a predictor of survival after heart transplantation [23]and survival of patients operated on due to non-small cell lung cancer (NSCLC) [24]. After exclusion of patients with significant hemorrhagic transformation, NLR remained significantly associated with cerebral edema and clinical end points [25]. As shown in the previous studies, NLR was closely associated with cardiovascular diseases, such as coronary artery disease (CAD) [26], HF [22], and heart transplantation [23]. The single measurement of NLR in patients admitted to the emergency department might be a useful tool for early identification of patients at high risk of acute kidney injury (AKI) [27]. Analogously, NLR was associated with the development of septic AKI and may have the potential for risk stratification of septic AKI [28]. The meta-analysis demonstrated that NLR is a predictor of all-cause mortality in patients with HF [29]. This study showed that NLR is an independent risk factor for all-cause and cardiac mortalities among patients with CAD over 5 years [26]. However, there are few researches on the relationship between NLR and ACS.

NLPR, a novel index, was derived from NLR. The derived inflammatory biomarker NLPR combined with platelets is a recognized hallmark of the development and progression of many diseases.

The NLPR is a reliable predictor of hemophagocytic lymphohistiocytosis associated AKI [10]. In chronic calculus cholecystitis (CCC), NLPR shows the best predictive power and specificity for predicting the severity of acute calculus cholecystitis (ACC) [12]. The NLPR in the carotid atherosclerosis group was significantly higher than those in the non-carotid atherosclerosis group. Besides, higher level of NLPR was associated with a higher risk of poor outcomes in patients with pyogenic liver abscess [30]. The value of NLPR in cardiovascular disease is also outstanding [31]. However, there are only few studies focusing on NLPR in patients with ACS undergoing PCI. The influence of the inflammatory index on cardiovascular diseases, especially ACS, has always been research hotspots. Previous studies have explored that various inflammatory indices, such as malondialdehyde (MDA), the systemic immune-inflammation index (SII, platelet × neutrophil/lymphocyte ratio), and the HALP (hemoglobin, albumin, lymphocyte, and platelet) score are predictive markers of cardiovascular disease [32–34]. 

Current researches have widely recognized the significance of inflammatory and nutritional indicators on the prognosis of patients with ACS. However, there are few studies combining different indicators for comprehensive evaluation in the clinical practice. Compared with single inflammatory indicators, the complex index can improve the diagnostic ability of coronary heart disease [21]. By combining SIRI with HDL, the derived SIRI/HDL index may enhance the diagnostic accuracy [21]. We used several approaches to investigate the correlation between NLPR, NLPR/HDL-C, NLPR×LDL-C, NLR, NLR/HDL-C, and NLR×LDL-C values and prognostic risk of ACS. The novel composite biomarkers, NLPR/HDL-C, NLPR×LDL-C, NLR/HDL-C, and NLR×LDL-C provide a more comprehensive assessment of systemic inflammation and lipid condition. Our study showed that NLPR and NLR, as novel indexes to predict the risk of MACEs, had the same efficiency as the former classical prognostic risk factors, which are the main factors affecting prognosis. We converted the continuous variables, considered as confounding factors in previous reports, into binary variables according to the standards of our hospital [35]. Variables with a P value less than 0.001 are considered to have more statistical significance, and they are selected into the model. The multivariate Cox proportional hazards model also demonstrated that NLPR/HDL-C ≥ 1.977, NLPR×LDL-C ≥ 4.608, NLR/HDL-C ≥ 0.030, and NLR×LDL-C ≥ 0.038 were independent risk factors for patients with ACS undergoing PCI. Interestingly, the time-ROC and RCS plots were used to analyze the correlations between NLPR, NLPR/HDL-C, NLPR×LDL-C, NLR, NLR/HDL-C, NLR×LDL-C, and MACEs. The time-ROC and RCS curves showed that the HR of patients with ACS undergoing PCI increased gradually with the NLPR, NLPR/HDL-C, and NLPR×LDL-C values.

In conclusion, NLPR ≥ 2.843, NLPR/HDL-C ≥ 1.977, NLPR*LDL-C ≥ 4.608, NLR ≥ 0.025, NLR/HDL-C ≥ 0.030, and NLR*LDL-C ≥ 0.038 were all independent prognostic risk factors in patients with ACS undergoing PCI, which may be useful markers for assessment for long prognosis. Therefore, patients with abnormal values of the above indicators may need more frequent follow-up to reduce the high risk of MACEs.

This study had some limitations. First, this was a single-center retrospective study with a small sample size. Second, NLPR, NLR, HDL-C, and LDL-C levels were evaluated at a single time point, and fluctuations in NLPR, NLR, HDL-C, and LDL-C levels were not considered. Finally, all enrolled participants were Chinese; therefore, the association between these inflammatory composite measures and long-term functional outcomes may need to be researched in populations from other countries.

## Conclusions

Higher NLPR, NLPR/HDL-C, NLPR×LDL-C, NLR, NLR/HDL-C, and NLR×LDL-C levels were independently associated with a higher risk of MACEs in patients with ACS undergoing PCI. The NLPR, NLPR/HDL-C, and NLPR×LDL-C had better predictive effects. The above indices of inflammatory and nutritional status may be novel biomarkers to assess the long-term prognosis in patients with ACS undergoing PCI.

## Supplementary Information

Supplementary Material 1.

## Data Availability

The raw data supporting the conclusions of this article will be made available by the authors without undue reservation.

## Abbreviations

NLPR: Neutrophil-to-lymphocyte*platelet ratio
NLR: Neutrophil-lymphocyte ratio
HDL-C: High-density lipoprotein cholesterol
LDL-C: Low-density lipoprotein cholesterol
MACEs: Major adverse cardiovascular events
ACS: Acute coronary syndrome
PCI: Percutaneous coronary intervention
HF: Heart failure
MI: Myocardial infarction
ISR: In-stent restenosis
KM: Kaplan– Meier
ROC: Receiver operator characteristic curve
AUC: Area under the curve
RCS: Restricted cubic spline
CAD: Coronary artery diseases
CVD: Cardiovascular disease
CHD: Coronary heart disease
LVEF: Left ventricular ejection fraction
