# Supplementary figures and images for: The complex inflammatory indexes predict the prognostic risk for patients with acute coronary syndrome undergoing percutaneous coronary intervention
Source: BMC Immunol. 2025 Sep 1;26:64. doi: 10.1186/s12865-025-00745-0 (PMC12400584; doi:10.1186/s12865-025-00745-0)

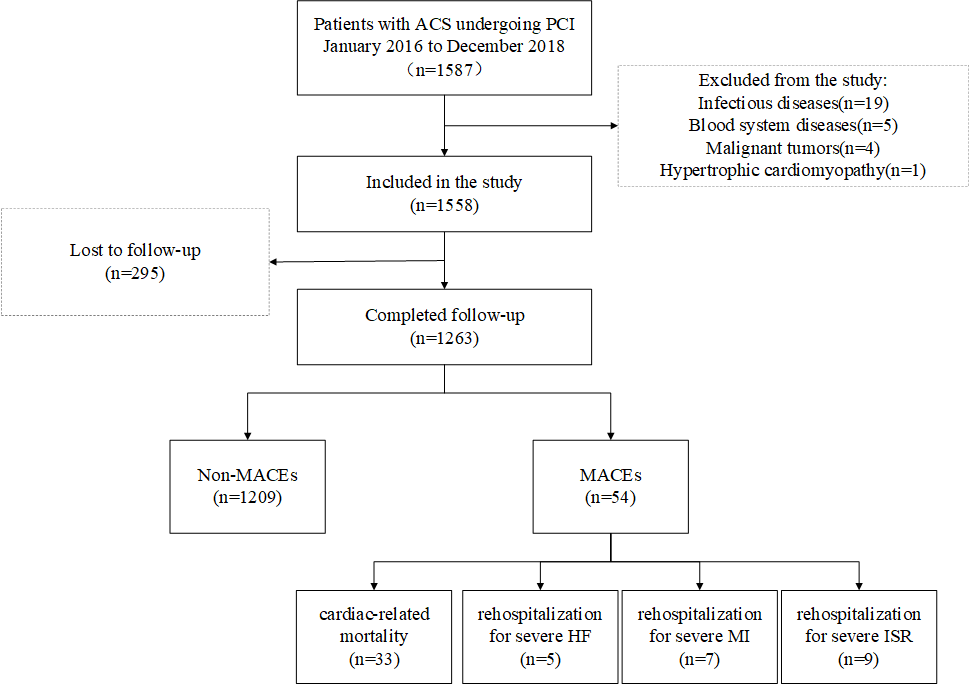

Supplement: Supplementary file 1 — Supplementary Material 1. [file 12865_2025_745_MOESM1_ESM.tif]
